# Temporal Differential Proteomes of *Clostridium difficile* in the Pig Ileal-Ligated Loop Model

**DOI:** 10.1371/journal.pone.0045608

**Published:** 2012-09-18

**Authors:** Tavan Janvilisri, Joy Scaria, Ching-Hao Teng, Sean P. McDonough, Robin D. Gleed, Susan L. Fubini, Sheng Zhang, Bruce Akey, Yung-Fu Chang

**Affiliations:** 1 Department of Population Medicine and Diagnostic Sciences, College of Veterinary Medicine, Cornell University, Ithaca, New York, United States of America; 2 Department of Biochemistry, Faculty of Science, Mahidol University, Bangkok, Thailand; 3 Institute of Molecular Medicine, National Cheng Kung University Medical College, Tainan, Taiwan; 4 Department of Biomedical Sciences, College of Veterinary Medicine, Cornell University, Ithaca, New York, United States of America; 5 Department of Clinical Sciences, College of Veterinary Medicine, Cornell University, Ithaca, New York, United States of America; 6 Proteomics and Mass Spectrometry Core Facility, Cornell University, Ithaca, New York, United States of America; Institute Pasteur, France

## Abstract

The impact of *Clostridium difficile* infection (CDI) on healthcare is becoming increasingly recognized as it represents a major cause of nosocomial diarrhea. A rising number of CDI cases and outbreaks have been reported worldwide. Here, we developed the pig ileal-ligated loop model for semi-quantitative analysis comparing temporal differential proteomes in *C. difficile* following *in vivo* incubation with *in vitro* growth using isobaric tags for relative and absolute quantification (iTRAQ). Proteins retrieved from the *in vitro* cultures and the loop contents after 4, 8, and 12 h *in vivo* incubation were subjected to in-solution digestion, iTRAQ labeling, two-dimensional liquid chromatography/tandem mass spectrometry and statistical analyses. From a total of 1152 distinct proteins identified in this study, 705 proteins were available for quantitative measures at all time points in both biological and technical replicates; 109 proteins were found to be differentially expressed. With analysis of clusters of orthologous group and protein-protein network interactions, we identified the proteins that might play roles in adaptive responses to the host environment, hence enhancing pathogenicity during CDI. This report represents the quantitative proteomic analysis of *C. difficile* that demonstrates time-dependent protein expression changes under conditions that mimic *in vivo* infection and identifies potential candidates for diagnostic or therapeutic measures.

## Introduction

*Clostridium difficile*, a gram-positive spore forming anaerobe, is an important nosocomial pathogen responsible for antibiotic-associated diarrhea. At present, the incidence and severity of *C. difficile* infection (CDI) worldwide is on the rise due to the increasing use of broad-spectrum antibiotics over the past decades and the emergence of hypervirulent antibiotic-resistant strains [1], [2]. *C. difficile* can infect humans as well as a wide variety of animals and possible transmission between species has been suggested [3]. Its ability to produce spores provides a potential source of infection and disease transmission. CDI leads to an array of disease conditions ranging from mild diarrhea to fatal pseudomembranous colitis [4]. It has been postulated that antibiotic treatment alters endogenous gut microflora, enabling *C. difficile* to colonize and produce toxins [5]. Both an enterotoxin (TcdA) and a cytotoxin (TcdB) enter the cells through colonic epithelial receptors. While TcdA promotes hyper-secretion of fluids and a subsequent inflammatory hemorrhage, TcdB causes cell death via alteration of the actin cytoskeleton [6]. The role of both TcdA and TcdB in CDI has been established [7], however, reports of CDI cases caused by *TcdA− TcdB+* strains [8] and contradicting experimental data suggest that only TcdB is essential for *C. difficile* virulence [9]. Certain *C. difficile* strains also produce a binary toxin, CDT, which enhances the formation of microtubule projection, thereby increasing the bacterial adherence and hence pathogenicity [10].

The first genome of *C. difficile* to be sequenced belongs to the strain 630, a virulent and multidrug-resistant strain isolated from a Swiss patient with severe CDI [11]. The genome sequences of two other strains, the non-epidemic strain CD196 and the hypervirulent strain R20291 have also been published and compared to that of 630, thereby identifying genetic markers that may play a part in hypervirulent traits [12]. Recently, an analysis of genetic variation among thirty *C. difficile* isolates from various PCR ribotypes through whole genome sequencing has been reported [13]. The genomes of other *C. difficile* strains have also been deposited in a public database (http://www.ncbi.nlm.nih.gov/sites/entrez?db=genome). Post-genomic investigations aim toward high-throughput data to further understand the mechanism of CDI. The use of microarrays for comparative genomic hybridization (CGH) between various *C. difficile* isolates and the reference strain 630 have revealed clade specificity and microevolution of hypervirulent strains [14] and gene conservation and divergence associated with host origin [15]. Integration of CGH data together with comparative genome sequencing and genome pathway analysis revealed that genome conservation in *C. difficile* is exceptionally low compared to other bacteria [16]. Furthermore transcriptomic analyses in *C. difficile* have been conducted under the environmental stresses and in the presence of antibiotics [17] as well as using chromosomal mutants [18]. Recently, we also reported the quantitative transcriptomes of *C. difficile* following the CDI *in vitro* [19] and *in vivo* [20]. Collectively, these studies pointed us toward the importance of additional factors other than toxins, which might be required for responses to environmental cues in the host surroundings and for regulation of virulence phenotypes.

Proteomic approaches have been developed into a powerful tool for generating high-throughput data to investigate several pathogenic bacteria [21]. The spore proteome of *C. difficile* 630 has been constructed based on 336 proteins identified by mass spectrometry and compared to the whole genome of other Clostridia [22]. Gel based approaches, e.g., with 2D-PAGE have been used to study cell surface proteins from *C. difficile* [23], [24]. The limitations of gel-based techniques include low levels of protein coverage and reproducibility [25]. In recent years, a non-gel-based quantitative shotgun proteomic approach using isobaric tags for relative and absolute quantification (iTRAQ) has been developed. The iTRAQ approach offers several advantages over the gel-based systems including the high level of proteome coverage, labeling efficiency, and accuracy in quantification [26], [27] and feasibility for simultaneously comparison of up to eight different experimental conditions in a single experiment [28]. In this work, we therefore utilized the iTRAQ technique to monitor proteomic changes in *C. difficile* grown *in vitro* compared to *in vivo* incubation in the pig ileal-ligated loops at different time points. A large-scale analysis of differential protein expression profiles together with functional category clustering and protein-protein network interactions would help us identify key proteins that could be of importance for successful host invasion during CDI and could lead to the identification of drug or vaccine targets.

## Materials and Methods

### Bacterial Culture

*C. difficile* strain 630 (kindly provided by Dr. Dale N Gerding) was grown anaerobically in pre-reduced brain-heart infusion (BHI) broth (Anaerobe Systems, Morgan Hill, CA) at 37°C for 12 h under anaerobic conditions. All experiments with anaerobic conditions were conducted in a Bactron IV anaerobic chamber (Shel Lab, Cornelius, OR) that was filled and purged with an anaerobic gas mixture (10% CO_2_, 85% N_2_, 5% H_2_). The chamber contained a catalyst, which removes any trace amounts of oxygen. All materials used in the anaerobic chamber were pre-reduced by incubation in the chamber for 24 h before use.

### Animal and Experimental Procedures

Pigs of both sexes and chronological age 8 weeks were obtained from Cornell University swine farm and were fed with weaner pellets and water *ad libitum*. In an attempt to mimic the progression of CDI, all the pigs received 3 doses daily of erythromycin and neomycin (50 mg each/kg body weight) for 3 days in a row, followed by 3 days in the absence of the antibiotics. The animals were fasted for 12 h prior to the surgery and were then delivered to the East Campus Research Facility at Cornell University. At the time of experiments, rectal swabs were cultured on blood agar plates at 37°C for 5 days in an anaerobic chamber and were tested for *C. difficile* toxin using Premier Toxin A&B ELISA kit following the manufacture’s protocol (Meridian Bioscience, Cincinnati, OH) to confirm the absence of *C. difficile* in the pigs. The animals were tranquillized with an intramuscular injection of xylazine (2 mg/kg body weight). Anaesthesia was induced and maintained with isoflurane. A 7-cm ventral midline celiotomy was performed and the intestine was exposed. Prior to the loop construction, the lumen of the ileum and distal jejunum was gently washed with phosphate-buffered saline (PBS) to remove the intestinal contents. For each pig, four 10-cm ligated small intestinal loops, starting with the ileum were formed with at least a 3-cm distance between loops using monofilament suture. *C. difficile* was grown to mid-log phase; the cells were pelleted and resuspended in the fresh pre-reduced BHI medium. Bacterial inocula containing 1×10^7^ cells in 10 ml pre-reduced BHI medium were injected into each of the intestinal loops of each experimental animal using a 21-gauge needle. One control loop injected with 10 ml of BHI medium was included for each animal as a control for host cell damage. Care was taken to avoid over-distension of loops. The abdominal incision was closed routinely and the animals were maintained under anesthesia until the end of the experiment. Based on the histopathological progression of host tissue damage, the inoculums were allowed to incubate *in vivo* in loop lumen for a period of 4, 8, or 12 h. After the incubation, the animals were sacrificed with an intravenous injection of pentobarbital sodium. The abdominal incision was opened and a segment of each loop was removed sequentially, in the same order in which they had been inoculated. The loop contents were then recovered and immediately placed on ice for protein extraction. Two experimental pigs were used for each time point. The swine husbandry followed the standard operating protocol CARE517, which can be found at http://www.research.cornell.edu/care/documents/SOPs/CARE517.pdf. All animals were cared for in compliance with the guide for the Care and Use of Laboratory Animals (US National Institute of Health) and the experimental animal handling was approved by the Institutional Animal Care and Use Committee (IACUC).

### Protein Extraction, Digestion and iTRAQ Labeling

To remove debris, the loop contents were centrifuged at 200×*g* for 10 min at 4°C. The supernatant was collected and subjected to further centrifugation at 12,000×*g* for 10 min at 4°C. To eliminate the possibility of host cell and debris contamination, percoll gradient centrifugation was performed. We also observed each fraction under microscope and the fractions that contained only intact cells were used to extract the total proteins. As the time required to complete the sporulation process is at least 24 h, we did not observe spores under microscope after the percoll centrifugation. A standard isoosmotic Percoll stock solution was prepared by diluting 90 ml of undiluted Percoll (Pharmacia, Inc., Piscataway, NJ) with 10 ml of 1.5 M PBS. A 60% (v/v) solution was made by combining 60 ml from the standard isoosmotic Percoll solution with 40 ml of 0.15 M PBS. The pellets were resuspended with PBS and were laid over 10 ml of 60% percoll in PBS. The mixtures were then centrifuged at 12,000×*g* for 45 min at 4°C. The upper fraction was discarded while the bacterial fractions were collected, washed with PBS and subjected to centrifugation at 40,000×*g* for 1 h at 4°C. The pellets were then resuspended with 10 mM PBS pH 7.4 containing 5% (v/v) protease inhibitor cocktail, 10 U of lysozyme and DNase and were incubated for 1 h at 37°C. The mixture was then subjected to centrifugation at 5,000×*g* for 10 min at 4°C. The pellets were resuspended in solubilization buffer containing 6 M Urea, 2 M Thiourea, 0.5% SDS, 0.5% CHAPS, 5 mM triethylammonium bicarbonate (TABC, pH 8.5). Ultrasonic disruption was performed on ice with bursts of 30 s at 40 kHz for 10 min (Sonifier SLPt, Branson, CT). Protein recovery was achieved by centrifugation at 21,000×*g* for 1 h at 4°C. Protein concentration was determined using Bradford assay with bovine serum albumin as a standard. A total of 100 µg protein of each sample was reduced with 5 mM tris[2-carboxyethyl] phosphine at 37°C for 1 h and the cysteine residues were blocked with 8 mM methyl methanethiosulfonate for 10 min at room temperature. The samples were then diluted to 1 M urea/0.08% SDS with 5 mM TABC prior to digestion with 10 µg of sequencing grade modified trypsin (Promega, Madison, WI) at 37°C for 16 h. The digested samples were completely dried in a SpeedVac (Thermo Savant, Holbrook, NY), and were reconstituted in 5 mM TABC buffer. Efficiency of protein digestion was assessed by SDS-PAGE using undigested and digested samples. Tryptic peptides from the control at the point of inoculations to the loops and *in vivo* incubation for 4, 8, and 12 h were labeled with iTRAQ reagents 114, 115, 116, and 117, respectively according to the manufacturer’s protocols (Document numbers 4351918A and 4350831C; which can be downloaded from http://docs.appliedbiosystems.com/search.taf; Applied Biosystems, Foster City, CA). The labeled samples were then combined and fractionated via a strong cation exchange chromatography (SCX) as described below.

### SCX Fractionation

SCX fractionation was completed using an Agilent 1100 HPLC with UV detector (Agilent Technologies, Santa Clara, CA). The iTRAQ-labeled tryptic peptides were reconstituted in 100 µl of buffer A (10 mM potassium phosphate pH 3.0, 25% acetonitrile; ACN), prior to SCX liquid chromatography. The samples (∼400 µg) were loaded onto a PolyLC polysulfoethyl A column (2.1 mm × 150 mm) purchased from PolyLC Inc. (Columbia, MD). Buffer B was composed of 10 mM potassium phosphate pH 3.0, 25% ACN with 1 M KCl. Sample fractionation was completed through the gradient of 0% buffer B for 15 min, 0–25% buffer B for 40 min, 25–50% buffer B for 10 min and hold in 50% buffer B for 10 min. During this elution, 40 fractions were collected at a flow rate of 200 µL/min on the basis of the UV trace at 214 nm. Several fractions were pooled post-collection to yield a total of 10 SCX fractions. Salts were removed via solid phase extraction using Waters SepPak C18 cartridge (Waters, Milford, MA) and purified peptide fractions were dried and reconstituted in 2% ACN, 0.05% formic acid for subsequent nanoLC-MS/MS.

### Nano-scale Reverse Phase Liquid Chromatography and Tandem Mass Spectrometry (nanoLC-MS/MS)

The nanoLC-MS/MS analysis was carried out using an UltiMate 3000 MDLC system (Dionex, Sunnyvale, CA) with a LTQ Orbitrap Velos mass spectrometer equipped with a nano ion source. A 5-µl aliquot of peptide fractions was injected onto a Symmetry C18 trapping column (300 µm×5 mm, 5 µm particle size; Dionex) at 10 µl/min flow rate for on-line desalting and then separated on a C-18 RP nanocolumn (75 µm×150 mm, 3 µm particle size) and eluted in a 90 min gradient of 2% to 38% ACN in 0.1% formic acid at 300 nl/min, followed by a 10-min ramping to 95% ACN-0.1% FA and a 5-min holding at 95% ACN-0.1% FA. The column was re-equilibrated with 2% ACN-0.1% FA for 20 min prior to the next run. The MS nano ion source contained a 10-µm analyte emitter (NewObjective, Woburn, MA). The Orbitrap Velos was operated in positive ion mode with the nanospray voltage set at 1.5 kV and the source temperature at 225°C. All data were acquired in a data dependent acquisition (DDA) mode using the Xcalibur 2.1 software (Thermo-Fisher Scientific). In DDA analysis, after each survey scan of the m/z ranging from 400 to 1,500, with detection in the Orbitrap mass analyzer at a resolution setting of 30,000, the 4 highest intensity ions with multiple charge states were selected for fragmentation at normalized collision energy of 45%.

### Data Processing, Protein Identification and Data Analysis

All MS and MS/MS raw spectra from iTRAQ experiments were converted to Mascot generic format using Proteome Discoverer 1.1 for subsequent database search using in-house licensed Mascot Deamon version 2.2.04 (Matrix Science, Boston, MA). The *C. difficile* 630 protein sequence database containing 3,753 sequence entries was downloaded from the NCBI reference sequence collection accession number NC_009089.1. The default search settings used for 4-plex iTRAQ quantitative processing and protein identification in Mascot server were as follows: one mis-cleavage for full trypsin with fixed MMTS modification of cysteine, fixed 4-plex iTRAQ modifications on lysine and N-terminal amines and variable modifications of methionine oxidation and 4-plex iTRAQ on tyrosine. The peptide mass tolerance and fragment mass tolerance values were 5 ppm and 0.8 Da, respectively. To estimate the false discovery rate (FDR) for a measure of identification certainty in each replicate set, an automatic decoy database search was performed in Mascot by choosing the decoy checkbox in which a random sequence of database is generated and tested for raw spectra along with the real database. To reduce the probability of false peptide identification, the significant scores for the peptides defined by a Mascot probability analysis (www.matrixscience.com/help/scoring_help.html#PBM) greater than “identity” were used as a filter and the resulting peptides were considered to be confidently-identified peptides and used for protein identifications. Furthermore, proteins identified in all four iTRAQ experiments, which contained at least two peptides with a *p*-value of <0.05 as determined by Mascot probability analysis were further analyzed. Intensities of the reporter ions from iTRAQ tags upon fragmentation were used for quantification. The relative quantitation ratios were processed with median normalization for each isobaric tag and rank normalization for the 4-plex in each set of experiments. A differentially expressed protein was defined as having (i) >1.5-fold difference as compared to the control, and (ii) a *p*-value of <0.05, based on t-distribution with Welch approximation. The heatmap and distance matrix were generated through the MultiExperiment Viewer (MeV) in the TM4 suite software [29]. Proteins were analyzed according to Clusters of Orthologous Groups (COGs) [30]. Known and predicted functional interaction networks of identified proteins were derived from the STRING database [31].

## Results and Discussion

### Overview of the Experimental Design

**Figure 1:**
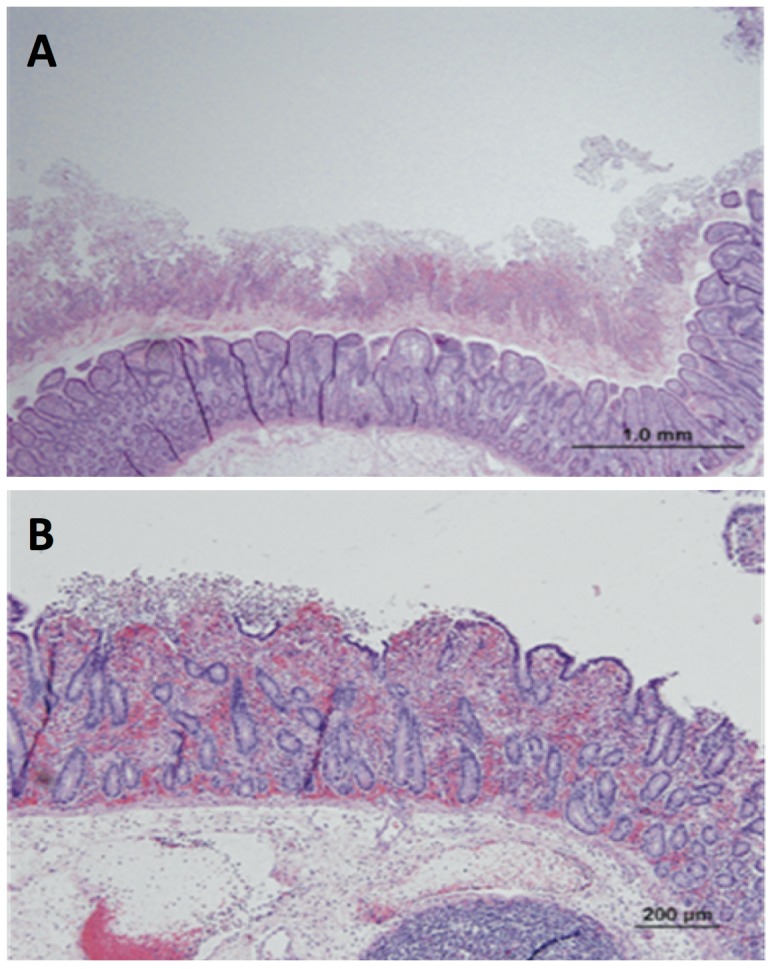
Microscopic sections of the ileal loops representing pseudomembrane formation due to the infection of *C. difficile* under the experimental conditions. (A) The mucosal surface is covered by a thick layer of fibrinopurulent exudate (‘pseudomembrane’). Note the extensive submucosal edema. (B) Focal erosion of the surface enterocytes is accompanied by exudation of fibrin and neutrophils into the lumen of the intestine.

To elucidate the pathogenic mechanism of *C. difficile*, it is important to understand how this pathogen adapts to the host environment. Here, we developed a pig ileal-ligated loop model and utilized proteomic techniques to study the differential proteome of *C. difficile* following the incubation *in vivo* compared to *in vitro* growth. The ileal loop model has previously been used to study the effects of TcdA, TcdB and CDT on rabbits [32]–[35]. These studies used either purified toxins or culture supernatants and only focused on the host pathophysiology. The piglet model of CDI has recently been reported, where the spores or vegetative cells of *C. difficile* were inoculated to the germ-free piglets to obtain the acute or chronic CDI characteristics. However, the number of intestinal *C. difficile* counts ranging from 0 to 10^11^ in small intestines is not correlated with the size of the inoculums and severity of disease [36]. In this study, our pig ligated-loop model allows the possibility to retrieve the bacterial cells and follow their proteome changes *in vivo*. We investigated the proteome of *C. difficile* at 4, 8, and 12 h following *in vivo* incubation compared with that of the *in vitro* culture. Representative microscopic sections of the ileal loops with pseudomembrane formation following the CDI are shown in Figure 1. The toxin concentration within the loop contents following the 4, 8, and 12 h incubation was estimated to 44.33, 109.5, and 146.0 ng/ml, respectively [20]. The histopathological progression of host tissue damage corresponded well with increased amounts of toxins in the loop contents. Although the loop model is well suited to characterize CDI, however limitations exist particularly when the bacteria present in the loop contents are compared to the *in vitro* culture. For examples, the pO_2_ within the loops may be different to standard BHI medium, the host uptake of nutrients may change the content of the medium, and also host factors may influence the growth of the pathogen.

**Figure 2:**
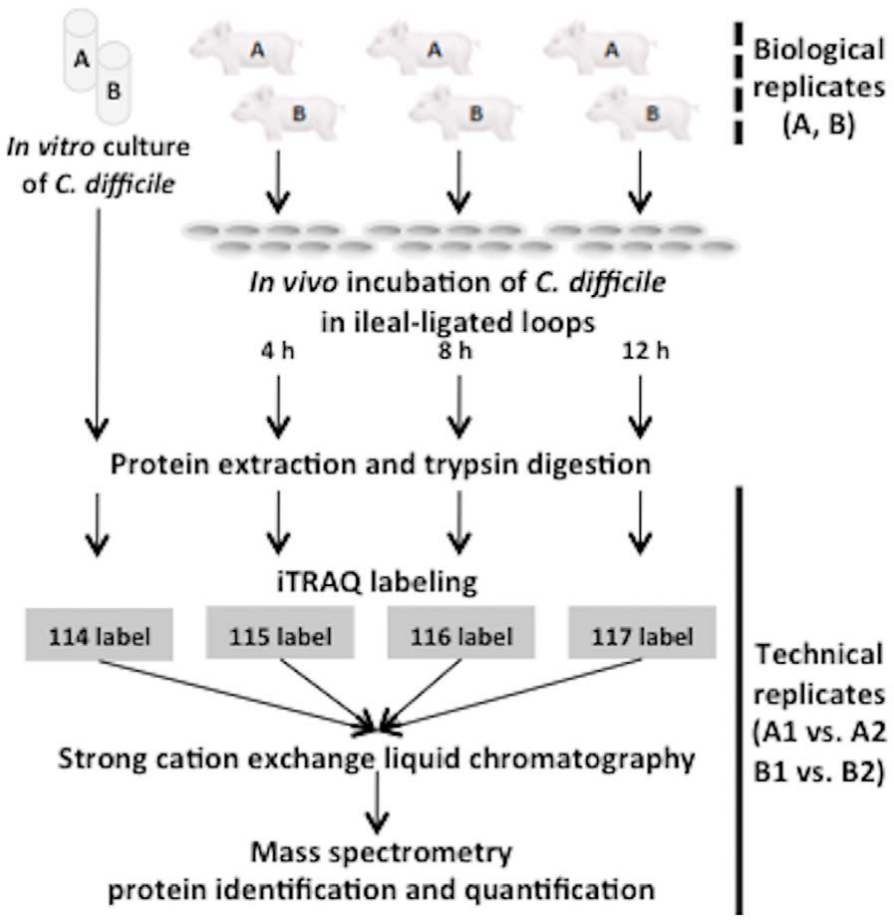
Overview of the experimental workflow. *C. difficile* retrieved from pig ileal-ligated loops following the *in vivo* incubation for a period of 4, 8 and 12 h were used for biological replicates (dotted line; A and B). The *in vitro* cultures of *C. difficile* were used as a control and subjected to the identical workflow. Proteins extracted from the control and the loop contents after 4, 8 and 12 h incubation were subjected to trypsin digestion and were labeled with the tags 114, 115, 116, and 117, respectively. Mass spectrometric results were analyzed for protein identification and quantification. Technical replicates were performed from the trypsin digestion step (solid line; A1 versus A2 and B1 versus B2).

An overview of the iTRAQ experimental strategy conducted in this study is shown in Figure 2. To reduce the possibility of false-positive protein identification and to control for biological, technical and experimental variations, four independent iTRAQ experiments were performed. Biological variation was controlled for at the culture and infection level from independent experiments (A and B) and technical variation was controlled including the protein digestion step, sample preparation and LC-MS/MS analysis (A1 vs. A2 and B1 vs B2). Proteins, whose expression levels were altered under *in vivo* incubation compared to the *in vitro* growth, were subsequently identified. Only proteins identified and quantifiable in all four iTRAQ experiments were further analyzed, allowing for stringent and sensitive protein identification and quantification of protein expression.

### iTRAQ Data Analysis

**Figure 3:**
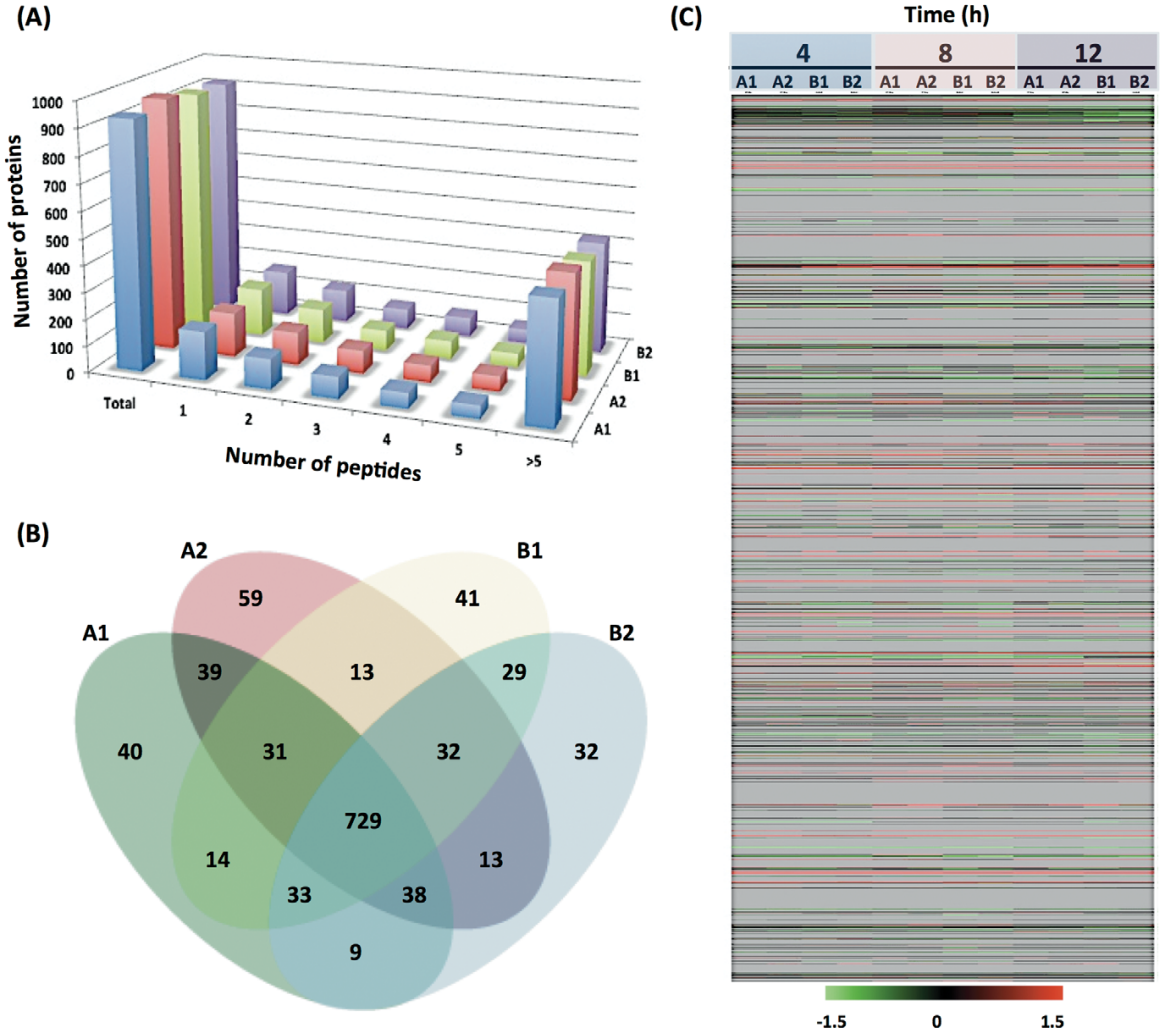
Overview of the iTRAQ results. (A) Number of distinct peptides identified in a protein. The peptides were identified using Mascot Daemon software with a 95% confidence level. About 17–19% of proteins were identified by single peptides. More than 80% of proteins were identified by at least two peptides. Approximately 47% of proteins were identified by more than 5 peptides. (B) Comparison of protein identification in iTRAQ experiments. Venn diagram shows the comparison of protein identification between biological replicates (A versus B) as well as technical replicates (A1 versus A2 and B1 versus B2). There were 207 proteins identified in a single experiment and 217 proteins identified in two or three of the experiments. A total of 728 proteins were identified in all four experiments. (C) Protein expression pattern in *C. difficile* following the *in vivo* incubation. Protein expression after 4, 8, and 12 h *in vivo* incubation is compared with that of *ex vivo* growth. Rank normalized data from biological and technical replicates were used to create a heatmap for *C. difficile*. The proteins were arranged according to the numbering of the *C. difficile* 630 coding sequences, with CD0001 at the top and CD3680 at the bottom, followed by proteins from the plasmid pCD630 (CDP01 to CDP11). Each column represents a particular time point in one iTRAQ experiment, and each row corresponds to a specific protein. The status of each protein is indicated by color as follows: red, induced; green, repressed; black, unchanged; and gray, undetected in our conditions.

Complete information with peptide identity filtered data sets for all proteins identified with a confidence interval of ≥95% in each individual iTRAQ experiment is provided in the File S1. The FDRs for the four sets of data are between 4.0–4.2% at 95% confidence interval, suggesting that the reported protein identifications were highly confident. To evaluate the reproducibility and effectiveness of the iTRAQ experiments, we compared the identified proteins from each iTRAQ set. Overall, 1,152 distinct proteins were detected with more than 95% confidence in all four iTRAQ sets. The numbers of proteins identified were 933, 954, 922 and 915 in A1, A2, B1, and B2, respectively. Figure 3A shows the number of peptides identified in a protein. Approximately, only 17–19% of proteins were identified with a single peptide. A total of 890 proteins (77.3%) were identified in both biological replicates (A and B). Technical replicates yielded 80.4–81.2% consistency (837/1,041 between A1 and A2; 823/1,014 between B1 and B2) in terms of protein identification. There were 728 proteins (63.2%) identified in all four experiments and only 207 proteins (18.0%) were unique for a single iTRAQ experiment (Figure 3B). Collectively, these results indicated the reliability of the iTRAQ protein identification of 728 proteins in all data sets. A total of 705 proteins were available for quantitative measures at all the time points in both biological and technical replicates and 109 proteins were considered to be differentially expressed, for which the criteria were that these proteins contained iTRAQ ratios from at least two peptides and exhibited at least a 1.5-fold difference compared to the control in at least one of the time points (*p*<0.05). The global protein expression profiles of *C. difficile* at 4, 8, and 12 h after *in vivo* incubation were compared with those of the *in vitro* cultured cells. A clear pattern of proteome regulation during the infection is presented as a heatmap in Figure 3C.

**Figure 4:**
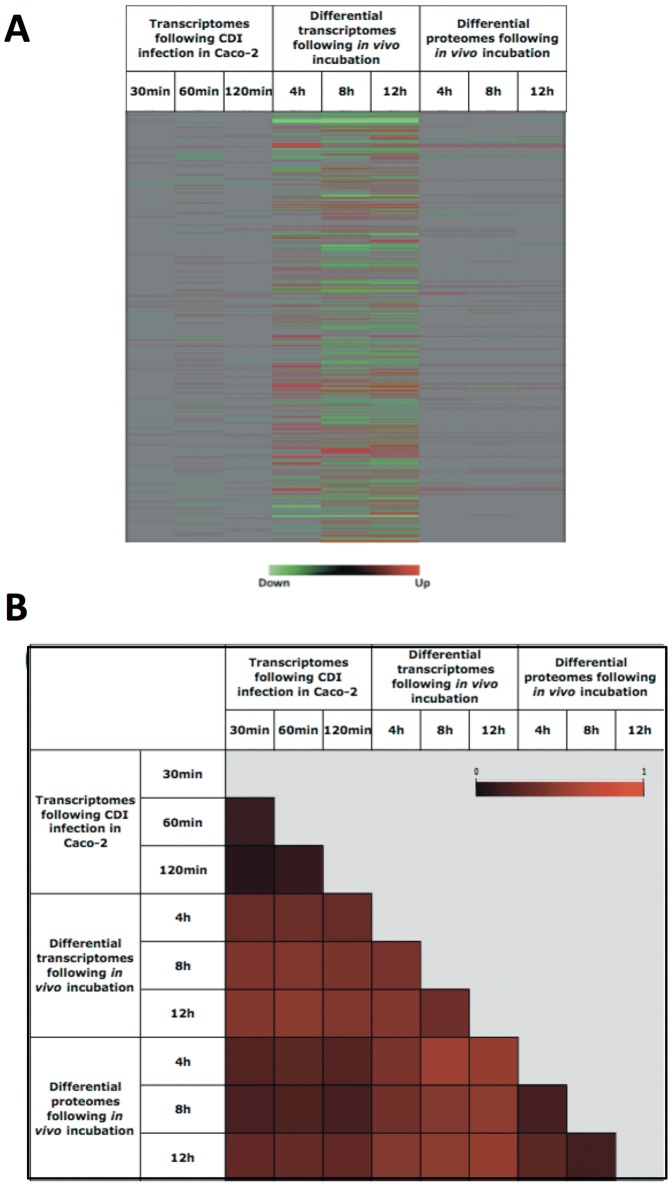
Comparison of the differential proteomes and transcriptomes during the CDI. (A) Heatmaps represent differentially expressed genes following the CDI in (i) Caco-2 cells (19) and (ii) pig ligated loop model (20), and (iii) differentially expressed proteins following the *in vivo* incubation in the ileal loops. Genes/proteins are ordered in rows beginning with CD0001 from the top down to CD3680, followed by CDP01-CDP11 at the bottom. Red and green colors indicate up- and down-regulation, respectively, compared to the control conditions. Grey denotes genes/proteins that are not differentially expressed. (B) Distance matrix represents the relationship between differentially expressed genes and proteins at different time points and conditions. Each square element within the matrix is rendered as a color that represents the distance between samples. A value of 0 indicates that two elements are identical whereas a value of 1 indicates that two elements are totally different. The main diagonal is simply rendered as white for identification.

We also compared the pattern of differential proteomes in this investigation to the differential transcriptome data from our previous studies, in which human colonic epithelial cells were infected with *C. difficile* 630 [19] and in which CDI was mimicked in the pig ligated loop model [20]. The patterns of gene/protein expression under different experimental settings are shown in Figure 4A. The distance matrices were also constructed to assess the relationship of the global expression patterns between each condition (Figure 4B). Comparison between the differential proteomes following the *in vivo* incubation and the differential transcriptomes following CDI *in vitro* and *in vivo* gave rise to scaled distance values ranging from 0.43–0.57 and 0.67–0.80, respectively. The variation of individual gene/protein expression under distinct experimental infection conditions was determined by the scaled distance values, which ranged from 0 to 0.645 with the average value of 0.264. These results revealed the high level of concordance between the proteomics and transcriptomics data. There are several possible explanations for the discrepancy between these data such as integrity of the transcripts, post-transcriptional modification, and technical limitations of the techniques.

### Functional Categories of Identified Proteins

**Figure 5:**
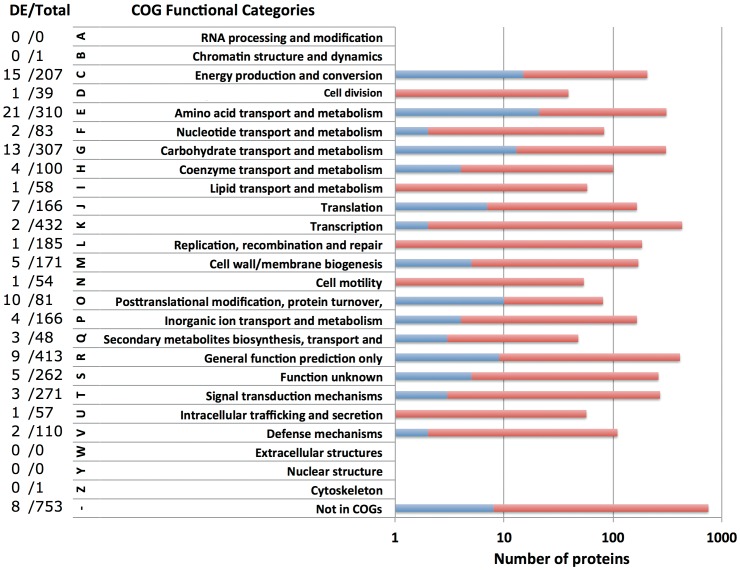
Distribution of differentially expressed proteins in *C. difficile* following *in vivo* incubation according to COGs functional categories. Of 3,753 proteins in *C. difficile* strain 630, there are 3,000 proteins found in COGs database. The stacked bar chart shows the percentage of differentially expressed proteins (blue) in each COGs functional category. The number of differentially expressed proteins and the total number of proteins in each COGs are also shown on the right panel. Of 109 differentially expressed proteins, 8 are not annotated in any COGs.

A total of 109 differentially expressed proteins were classified using an enrichment analysis based on the COGs. The analysis of the abundance of each COGs functional category revealed substantial differences in *C. difficile* proteome upon *in vivo* incubation. The percentage of each COG’s functional class of differentially expressed proteins relative to the total predicted proteome is schematically depicted in Figure 5. Overall, ∼40.7% of the identified proteins were annotated as being involved in metabolic processes including metabolism of amino acids and their derivatives (17.8%), carbohydrates (11.0%), coenzymes (3.4%), inorganic ions (3.4%), nucleic acids (1.7%), and lipids (0.8%). These results are in agreement with the previous data where the differentially expressed genes involved in metabolism were most prevalent following the CDI *ex vivo* (19) and *in vivo* (20). A significant number of proteins belonged to the predicted functional roles corresponding to energy production and conversion (12.7%), posttranslational modification (8.5%), translation (5.9%), biogenesis of cell wall and plasma membrane (4.2%), signal transduction mechanisms (2.5%), and transcription (1.7%). Proteins associated with replication, recombination, repair, biosynthesis of secondary metabolites, intracellular trafficking, cell cycle and cell motility were also identified during the infection (Figure 5). Furthermore, 9/109 COGs from differentially expressed proteins were associated with general function prediction only, and 5/109 were related to unknown function, while 8 differentially expressed proteins were not assigned for COGs.

Table S1 lists the details of 109 differentially expressed proteins identified in this study according to their biological roles with the fold changes at each time point following the *in vivo* incubation compared to the *in vitro* growth. Comparison of these proteins to the *C. difficile* 630 spore proteome [22] revealed that 26/336 (7.7%) spore-associated proteins were found to be differentially expressed during CDI *in vivo*. These proteins mainly function in translation, posttranslational modification, energy production and metabolism, indicating that these processes are essential to both spore germination as well as virulence during CDI. Interestingly, 17/336 essential proteins functioning in sporulation/germination from the spore proteome were not detected in our experimental conditions. To gain an overview of the biological interaction among the identified proteins, we constructed the protein-protein functional networks using String database (Figure S1). The protein network analysis provides us a clearer view of a complex framework of proteins that play roles in host adaptation and pathogenesis. The following sections discuss in detail the proteins identified in this study in response to the *in vivo* incubation compared to the *in vitro* growth conditions according to their functional categories.

### Metabolic Processes and Energy Production

Many proteins facilitating key steps in metabolic processes were found to be differentially-expressed. Among others, proteins involved in the succinate fermentation to butyrate pathway including AbfD (CD2341), SucD (CD2342), and Cat1 (CD2343) showed a decrease in abundance whereas proteins in pyruvate fermentation to acetate pathway including Pyc (CD0021), and PflD (CD3282) were up-regulated. Interestingly, expression of the proteins in proline interconversion pathway such as PrdF (CD3237), PrdB (CD3241), PrdA (CD3244) and ProC2 (CD3281) as well as proteins in histidine metabolism including HisC (CD1549), HisB (CD1550), HisA (CD1552), HisF (CD1553), and HisI (CD1554) were induced (Table S1). Since *Clostridium* species can utilize combinations of amino acids for growth through fermentations of amino acid pairs in which one amino acid is oxidized and the other one is reduced, our findings suggest that *C. difficile* may adapt to the host environment, where energy sources are limited, by shifting the energetic and metabolic resource to maintain an optimal growth. Interestingly, proteins involved in sulfur relay system including cysteine desulfurase IscS2 (CD1279), iron-sulfur binding protein (CD2169), and anaerobic sulfite reductase subunits AsrA (CD2233) and AsrB (CD2232) were found to be up-regulated at all-time points *in vivo* compared to the control. Sulfite reduction is the central energy-conserving step of sulfate respiration [37]. In environments devoid of oxygen, the ability to use sulfate as a terminal electron acceptor for anaerobic respiration plays an essential role and suggests a preference for energy production during CDI. Sulfur reduction is also crucial for the production of cysteine derivatives. The existence of links between expression of *C. difficile* toxin and cysteine derivatives has been shown [38], suggesting the possibility of the relevance of sulfur metabolism towards *C. difficile* virulence during CDI.

### Cellular Processes and Signaling

A number of proteins involved in regulatory functions, signal transduction, and protein folding/processing exhibited differential abundance as a result of the *in vivo* incubation. Among these, the induction of thioredoxin TrxA1 (CD1690) and thioredoxin reductase TrxB1 (CD1691) were observed. Thioredoxins are small electron-transfer proteins that contain a cysteine disulfide/dithiol active site and serve as the major cellular protein disulfide reductases. TrxA1 is reduced by NADPH in a reaction catalyzed by TrxB1. The conversion between the oxidized and reduced forms results in a change of conformation and a significant change in functional properties. The reduced thioredoxin is a powerful protein disulfide reductase that catalyzes dithiol-disulfide exchange reactions with many substrates [39]. These proteins may serve as the molecular responses to a reducing environment, hence play a significant role in defending the cell against oxidative stress, confirming the involvement of sulfur metabolism in *C. difficile* pathogenesis. Furthermore, the chaperonin (HslO), the ATP-dependent Clp protease, proteolytic subunits B (ClpB; CD2020) and C (ClpC; CD0026) were up-regulated. The protein expression of chaperone DnaJ (CD2460) slightly increased after 4-h *in vivo*, but became reduced by 12-h. In general, the molecular chaperones play a role in ATP-mediated protein folding, and the Clp proteins proteolytically degrade misfolded proteins [40]. These clusters of proteins act as protein quality control systems in response to protein folding stress that might be induced by the environmental host conditions or the defense mechanism [41].

While some proteins involved in carbohydrate utilization through phosphotransferase systems (PTS) including PtsG (CD2667), MalX (CD3030), and CD3137 were down-regulated, others including CD0287, GatC (CD2325), PtsH (CD2756), CD3068, and CD3069 were found to be up-regulated. These results suggest that the changes in metabolic states of *C. difficile* within the host environments may provide another mechanism for host adaptation when the nutrient source might be scarce. There is also evidence that the PTS relate to virulence traits [42], where shifts in carbon source availability can trigger virulence gene regulators, therefore differential PTS protein expression may contribute to *C. difficile* pathogenicity. Although below the cutoff point, the transcriptional repressor CodY (CD1275) was found to be significantly up-regulated with the fold difference of 0.95, 0.56, and 0.12 at the time of 4 h, 8 h, and 12 h following the CDI, respectively. The results corresponded well to the *CodY* transcripts in the pig ileal loops were found to be induced ∼4-fold at 4 and 8 h [20]. The posttranslational modification and degradation of this protein may explain the differences in the level of induction found in these settings. It has been reported that its active form is necessary for repression of toxin genes in *C. difficile* [43], [44]. In fact, the CodY expression pattern is correlated well with the amount of total toxins measured by ELISA, where the highest toxin concentration was observed at 12 h [20].

The expression of certain cell surface proteins including flagellar protein FliS1 (CD0235), putative CDP-glycerol: Poly(glycerophosphate) glycerophosphotransferase (CD0244), and putative N-acetylmuramoyl-L-alanine amidase (CD1035), were found to be reduced. Mutagenic analysis of the *C. difficile* flagellar proteins revealed that the absence of flagella increases the bacterial capability for colonization and adherence to host tissues [45]. Other proteins involved in cell envelope formation including rod shape-determining protein MreB1 (CD0127), CD1047, CD1898, and CD2796 were up-regulated. Altogether, as the surface proteins facilitate cell-cell adhesion and stabilization of the membrane [46], it may be possible that changes in the protein abundance provide a common strategy for remodeling bacterial cell walls to adapt to changing environmental conditions [47]. On the other hand, the bacterial surface proteins protect the cells from mechanical stress [46] and the changes in their abundance may be as a result of the host response to CDI.

For the transport and binding functional category, the expression of RbsB (CD0300), RbsA (CD0301), PotA (CD1024), FeoB1 (CD1479), MetQ (CD1491), CD1618, CD2365 were elevated whereas the expression of proteins in the gene cluster CD0873, CD0874, and CD0875 were suppressed. Interestingly, CD2169 was up-regulated from 2.5- to 3.2-fold following the *in vivo* incubation. The protein expression corresponded well to its mRNA levels [20]. It is the P-loop ATPase containing an inserted ferredoxin domain, which can mediate electron transfer in a range of metabolic reactions, supporting our results that hinted at the involvement of sulfur metabolism during CDI. However, experimental evidence is certainly needed to verify the hypothesis.

### Transcription and Translation

Certain proteins responsible for transcription and translation showed differential abundance following the infection (Table S1). We observed down-regulation of 5 proteins involved in protein synthesis following the *in vivo* incubation. They belonged to the group of ribosomal proteins such as RpmG (CD0058A), RpsN (CD0084A), RplU (CD1161), RpmF (CD1176A), and RpmE (CD3486A) that formed a tight interaction network as seen in the protein-protein interaction map (Figure S1). Since genes encoding ribosomal proteins in *C. difficile* are clustered into operons with various sizes, therefore the down-regulation of these proteins may represent the down-regulation of operons as a whole. Consistent with the proteomic data, our transcriptomic study revealed that the genes encoding ribosomal proteins were also repressed upon the *in vivo* incubation [20], however the level of ribosomal protein transcripts were induced under *in vitro* setting of CDI [19], pointing to differential modulation of transcriptional and translational pathways under *ex vivo* and *in vivo* conditions. Interestingly, the catabolite control transcriptional regulator CcpA (CD1064) and the heat-inducible transcriptional repressor HrcA (CD2463) were found up-regulated at 4 h and the degree of induction decreased in a time-dependent fashion. It has been reported that CcpA binds to the regulatory region of *tcdA* and *tcdB* genes, thereby repressing toxin expression in response to PTS sugar availability [48], thus confirming the link between carbon source utilization and virulence gene expression in *C. difficile*. Altogether, these data may hint to the bacterial mechanism response to the stress in host environment.

### Unknown Functions and Hypothetical Proteins

In addition to the proteins discussed above, much of the response of *C. difficile* 630 to a host environment is regulated via proteins with unknown functions and hypothetical proteins. Although the function of certain hypothetical proteins has been predicted by domain homology searches and has been classified according to COGs (Table S1), there are still a large number of hypothetical proteins with no homology to other proteins in the database, but they are highly conserved within the species. Of the 341 hypothetical proteins in the genome of *C. difficile* 630, we identified 10/341 (2.9%) differentially expressed hypothetical proteins in this study. Among these, only one was down-regulated, while 9/10 were up-regulated. The maximum fold difference was found in CD0279 whose expression was up-regulated at all time points up to 7.5-fold at the time of 4 h *in vivo*. CD0279 is found to be highly conserved with hypothetical proteins in *C. difficile* NAP07 (ZP_06904903.1), NAP08 (ZP_06894325.1), CD196 (YP_003213345.1), R20291 (YP_003216791.1), ATCC43255 (ZP_05349479.1), CIP107932 (ZP_05320810.1), QCD32g58 (ZP_07405305.1), QCD-23m63 (ZP_05399754.1), QCD-63q42 (ZP_05328426.1), QCD-66c26 (ZP_05270418.1), QCD-76w55 (ZP_05354573.1), QCD-97b34 (ZP_05383425.1), and QCD-37x79 (ZP_05395744.1). These results highlight the fact that the processes in *C. difficile* pathogenesis and host adaptation remain highly uncharacterized and we cannot overlook the roles of these proteins, which might lead us to novel targets for diagnostic and therapeutic purposes. To assign their biological functions, further investigation is needed. The identification of these hypothetical proteins unique to this work will certainly lead us as well as others in the field towards further studies on their biological relevance to *C. difficile* pathogenesis.

### Conclusions

In this work, the quantitative proteome profile of *C. difficile* was generated using the iTRAQ labeling. The iTRAQ method provides a valuable and reliable tool in the analysis of differential global protein expression in complex samples such as the ones studied here. The differential proteomes of *C. difficile* following the *in vivo* incubation in the pig ileal-ligated loop model, which was designed to mimic the CDI, were investigated. We identified 109 proteins that were differentially expressed following *in vivo* incubation. Our results showed that there was a general trend of overall decrease in protein expression when *C. difficile* was exposed to the host environment compared to cell grown *in vitro*. The down-regulation of certain proteins involved in metabolism and energy production can be interpreted as the cellular response to limited nutrient conditions within the host. The identification of proteins unique to this work shed light on their biological relevance to *C. difficile* pathogenesis, especially bacterial adaptation to host environments during CDI. As data on transcriptomes and proteomes of *C. difficile* accumulate, further analyses will help complete the cellular pathways. Future endeavors aiming to investigate the molecular and cellular function of these proteins will surely advance our understanding of the complex mechanisms of this important pathogen.

## Supporting Information

Figure S1 **Functional interaction network of differentially expressed proteins in** ***C. difficile*** **following the** ***in vivo*** **incubation.** Nodes are either colored (linked) or white (nodes of a higher iteration/depth). Edges, i.e. known and predicted functional links consist of recurring neighborhood (green), gene-fusion events (red), phylogenetic co-occurrence (blue), co-expressions (grey), biochemical data (pink), evidence from databases (light blue) and text mining (yellow).
(TIFF)

Table S1 **A list of differentially expressed proteins identified in this study and their expression levels in response to** ***in vivo*** **conditions compared to the** ***in vitro*** **growth.** The positive and negative values of the means represent up- and down-regulation, respectively. These proteins are classified according to their clusters of orthologous groups.
(XLSX)

File S1 **All protein and peptide data are available as spreadsheets.** The tables list the identified proteins with their highest scoring peptides with the following information from left to the right: Hit number according to MASCOT scoring (prot_hit_num), protein accession number, protein name (prot_desc), MASCOT protein score (prot_score), M.W. [Da] (prot_mass), total numbers of peptides sequenced (pep_matches), peptide coverage (prot_cover), protein pI (prot_pi), scan number (pep_query), highest scoring peptide (pep_rank), bold red peptide (pep_isbold), experimental m/z value (pep_exp_mz), experimental M.W. (pep_exp_mr), charge state (pep_exp_z), calculated M.W. (pep_calc_mr), mass deviation (pep_delta), number of missed cleavages (pep_miss), highest peptide score according to MASCOT (pep_score), peptide score homology threshold (pep_homol), peptide score identity threshold (pep_ident), peptide expectation value according to MASCOT (pep_expect), amino acid C-terminal to tryptic peptide (pep_res_before), peptide sequence (pep_seq), amino acid N-terminal to tryptic peptide (pep_res_after), variable modification (pep_var_mod), position of variable modfcation (pep_var_mod_pos).
(XLSX)
