# Supplementary figures and images for: Temporal Differential Proteomes of Clostridium difficile in the Pig Ileal-Ligated Loop Model
Source: PLoS One. 2012 Sep 18;7(9):e45608. doi: 10.1371/journal.pone.0045608 (PMC3445491; doi:10.1371/journal.pone.0045608)

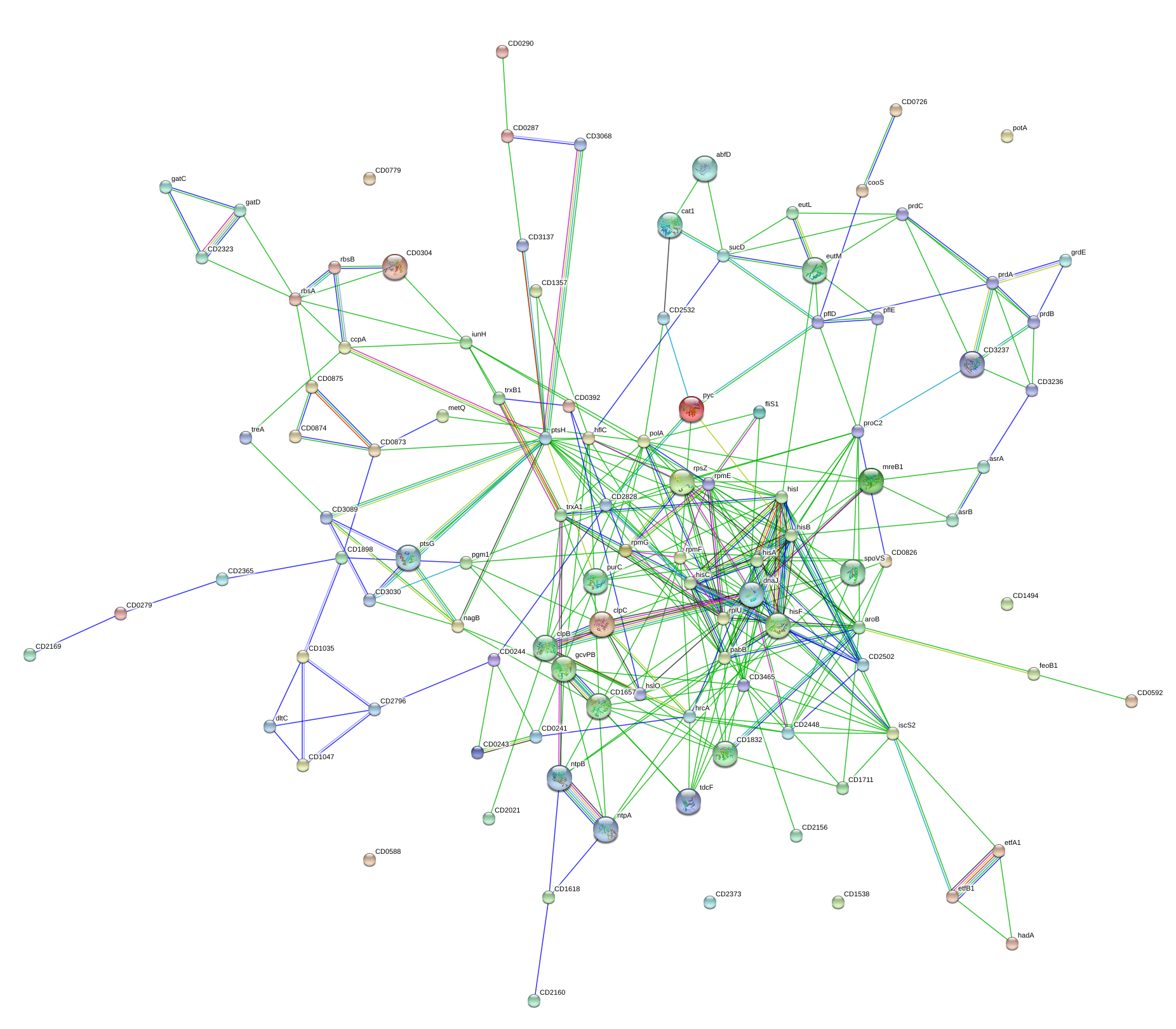

Supplement: Figure S1 — Functional interaction network of differentially expressed proteins in C. difficile following the in vivo incubation. Nodes are either colored (linked) or white (nodes of a higher iteration/depth). Edges, i.e. known and predicted functional links consist of recurring neighborhood (green), gene-fusion events (red), phylogenetic co-occurrence (blue), co-expressions (grey), biochemical data (pink), evidence from databases (light blue) and text mining (yellow). (TIFF) [file pone.0045608.s001.tiff]
